# Impaired Volume Regulation and Electrophysiology of Astrocytes In Situ in a Mouse Model for Megalencephalic Leukoencephalopathy With Subcortical Cysts

**DOI:** 10.1002/glia.70047

**Published:** 2025-05-30

**Authors:** Sven Kerst, Nina Meesters, Tim S. Heistek, Marjo S. van der Knaap, Huibert D. Mansvelder, Rogier Min

**Affiliations:** ^1^ Department of Child Neurology Amsterdam Leukodystrophy Center, Emma Children's Hospital, Amsterdam University Medical Center, Amsterdam Neuroscience Amsterdam the Netherlands; ^2^ Department of Integrative Neurophysiology Center for Neurogenomics and Cognitive Research, Vrije Universiteit Amsterdam, Amsterdam Neuroscience Amsterdam the Netherlands; ^3^ Department of Neurology, Section of Epileptology, Uniklinik RWTH Aachen Aachen Germany

**Keywords:** astrocyte, electrophysiology, GlialCAM, leukodystrophy, volume regulation, white matter edema

## Abstract

Electrical signaling, driven by ion fluxes between intra‐ and extracellular compartments, is central to brain functioning. Astrocytes provide crucial support by maintaining the homeostasis of water and ions in the brain. This is disrupted in the leukodystrophy Megalencephalic Leukoencephalopathy with subcortical Cysts (MLC). Studies on cultured primary astrocytes and other isolated cell lines point to a central defect in astrocyte volume regulation in MLC. However, cell culture severely alters the properties and polarity of astrocytes. Therefore, whether astrocytes in the intact MLC brain exhibit aberrant physiology related to water and ion homeostasis remains unknown. To investigate astrocyte physiology in intact astrocytes, we performed experiments in acute brain slices from a validated MLC mouse model, the *Glialcam*‐null mouse. We combined viral sensor delivery with two‐photon microscopy to study astrocyte volume regulation and associated chloride dynamics. Cortical *Glialcam*‐null astrocytes showed normal intracellular chloride dynamics but reduced volume recovery upon potassium‐induced cell swelling. Whole‐cell patch‐clamp recordings revealed a modestly depolarized resting membrane potential and slower glutamate uptake in *Glialcam*‐null astrocytes. Gap junction coupling of the astrocyte syncytium was modestly reduced, but it remained sufficient to preserve functional electrical isopotentiality. In conclusion, our findings confirm that the previously observed disturbance of astrocyte volume regulation observed in cultured cells is also observed in intact astrocytes in situ, and we uncover additional changes in astrocyte electrophysiological properties. These findings support the concept that dysfunctional astrocyte volume regulation is central to the MLC disease mechanism.

## Main Points

- Cell culture studies show disrupted astrocyte physiology in the leukodystrophy MLC. Are intact astrocytes similarly affected?
- This study on astrocytes in MLC mouse brain slices confirms and extends in vitro studies on altered astrocyte physiology.

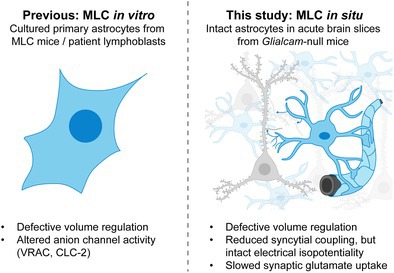

## 1 Introduction

Astrocytes play a vital role in maintaining neuronal network homeostasis by modulating synaptic connectivity, neuronal excitability, and brain‐wide fluid dynamics (Verkhratsky and Nedergaard 2018). Disruption of astrocyte‐mediated homeostasis has been implicated in various neurological diseases (Min and van der Knaap 2018). An astrocytic defect is central in the leukodystrophy Megalencephalic Leukoencephalopathy with subcortical Cysts (MLC). MLC is characterized by chronic white matter edema, extensive myelin vacuolization, progressive motor dysfunction, and mild cognitive impairments (Hamilton et al. 2018). Epileptic seizures are also common in MLC patients.

Most MLC cases are linked to pathogenic variants in *MLC1* or *GLIALCAM*. Mouse models deficient in these genes (*Mlc1*‐null mice and *Glialcam*‐null mice) replicate the characteristic early‐onset intramyelinic edema in MLC and provide a useful model to study the cellular pathophysiology of MLC in intact tissue (Bugiani et al. 2017; Dubey et al. 2015). MLC1 and GlialCAM, which tightly interact, are enriched in astrocyte endfeet, where they interact with many different transporters and ion channels (Bosch and Estevez 2020; Capdevila‐Nortes et al. 2013). These include the K_ir_4.1 potassium channel and the Na^+^/K^+^‐ATPase, which regulate potassium homeostasis and indirectly modulate glutamate uptake (Capuani et al. 2016; Romanos et al. 2020). Studies in acute brain slices from both *Mlc1*‐null mice and *Glialcam*‐null mice have demonstrated impaired regulation of extracellular potassium and glutamate, mechanisms thought to contribute to the seizure phenotype in MLC (Dubey et al. 2018; Kater et al. 2023).

The glutamate and potassium clearance capacity of astrocytes relies on extensive gap junction coupling of these cells. Astrocytes and oligodendrocytes form the so‐called panglial syncytium. Syncytial coupling allows long‐distance dispersal of water and ions and helps astrocytes to maintain electrical isopotentiality (Ma et al. 2016). In cultured cells, GlialCAM directly interacts with the gap junction protein connexin‐43 (Cx43). GlialCAM might therefore influence the panglial syncytium (Wu et al. 2016); however, gap junction coupling and syncytial isopotentiality of astrocytes in intact MLC mouse brain tissue have not been studied. The active uptake of ions and osmolytes leads to the influx of osmotically obliged water, which causes astrocyte swelling (Wilson and Mongin 2018). Therefore, another important factor relevant to clearance capacity is the ability of astrocytes to counteract swelling by regulating their volume. Lymphoblasts derived from MLC patients and cultured primary astrocytes from *Mlc1*‐null mice show defects in swelling‐activated volume‐regulated anion channel (VRAC) function, leading to a defect in regulatory volume decrease (RVD) (Dubey et al. 2015; Passchier et al. 2023; Ridder et al. 2011). Cultured rat astrocytes with hampered *Glialcam* expression have a similar VRAC activation defect (Capdevila‐Nortes et al. 2013). Therefore, studies in cell culture suggest several alterations in astrocyte physiology that could underlie disturbed potassium and glutamate homeostasis in MLC. However, cultured astrocytes are disconnected from their neuronal environment and from the circulation and lose their complex morphology and characteristic polarization. It is therefore crucial to study the extent to which observations on astrocyte physiology in culture translate to astrocytes in situ (astrocytes in their natural configuration in the brain). Moreover, whether MLC astrocytes in situ have other disturbed electrophysiological properties that could lead to network hyperexcitability in MLC remains unknown.

Here, we studied astrocyte volume regulation and electrophysiological properties in acute brain slices of *Glialcam*‐null mice. We show that *Glialcam*‐null astrocytes in situ are impaired in their volume regulation upon physiologically relevant cell swelling, without showing significant alterations in accompanying chloride dynamics. At the electrophysiological level, we find that *Glialcam*‐null astrocytes have a depolarized resting membrane potential and a reduced glutamate uptake capacity. While gap junction coupling between astrocytes is modestly impaired, this does not alter the electrical isopotentiality of the astrocyte syncytium in *Glialcam*‐null mice. These results provide important insights into how astrocytic defects may lead to network hyperexcitability and loss of homeostatic support in MLC.

## 2 Materials and Methods

### 2.1 Animals

*Glialcam* deficient (*Glialcam*‐null) mice were generated as previously described (Bugiani et al. 2017; Dubey et al. 2018; Favre‐Kontula et al. 2008) and maintained on a C57Bl6 background. *Glialcam*‐null mice were compared to wild‐type littermates. All experiments were approved by the Animals Ethical Committee of VU University in Amsterdam, in accordance with Dutch law.

### 2.2 Virus Injection

To visualize astrocyte chloride dynamics, we used the chloride sensor SuperClomeleon (SC) (Grimley et al. 2013) delivered with an AAV2/5 viral vector under the GfaABC_1_D promoter (Shigetomi et al. 2013). The AAV2/5‐GfaABC_1_D::SC vector (SC plasmid from the lab of Prof. George J. Augustine, Nanyang Technological University, Singapore) was produced by VectorBuilder (ID #VB190703‐1052azz, titer 4.75 × 10^13^ GC/mL). Thirty minutes before SC virus injection surgery, mice received 0.1 mg/kg Temgesic (RB Pharmaceuticals, UK). Mice were anesthetized with isoflurane or with an intraperitoneal (i.p.) injection of a mixture of 0.05 mg/kg fentanyl (Fentadon; Dechra, UK), 5 mg/kg midazolam (Midazolam; Actavis, Ireland) and 0.5 mg/kg medetomidine (Sedastart; ASTfarma, the Netherlands) and placed onto a stereotaxic frame. For local analgesia, lidocaine (2%, Sigma‐Aldrich Chemie N.V, the Netherlands) was applied on the surface of the skull. The viral vector was injected bihemispherically in the upper layers of the primary visual cortex (AP: −4.04 mm, ML: ±2.5 relative to Bregma, DV: −0.1 mm relative to pia). Approximately 150 nL of virus was injected per site at a speed of 23 nL/s using a Nanoject II (Drummond SCI, USA). The needle was left in place for 5 min following injection. In case injection anesthesia was used, mice were injected (i.p.) with a mixture of 1.2 mg/kg naloxone (Naloxon HCL‐hameln; Hameln Pharma Plus GmbH, Germany), 0.5 mg/kg flumazenil (Flumazenil Kabi, Fresenius Kabi, Germany), and 2.5 mg/kg atipamezole (Sedastop; ASTfarma, the Netherlands) to counteract anesthesia after surgery.

### 2.3 Acute Brain Slice Preparation

For two‐photon imaging of SC‐expressing astrocytes in acute brain slices, 3‐ to 5‐month‐old mice were used, ≥ 2 weeks after virus injection. For electrophysiological recordings, 2‐ to 4‐month‐old mice were used. Following decapitation, brains were removed and placed in an ice‐cold cutting solution, containing (in mM): 93 NMDG, 2.5 KCl, 1.2 NaH_2_PO_4_, 30 NaHCO_3_, 20 HEPES, 10 MgSO_4_, 0.5 CaCl_2_, 25 d(+)‐glucose, 3 Na‐pyruvate, and 5 Na‐ascorbate (osmolality 305 mOsm; carboxygenated with 95%O_2_/5%O_2_). Coronal slices (300 μm), including the primary visual cortex, were made with a vibratome. Slices were kept in room temperature holding solution, containing (in mM): 93 NaCl, 2.5 KCl, 1.2 NaH_2_PO_4_, 30 NaHCO_3_, 20 HEPES, 10 MgSO_4_, 0.5 CaCl_2_, 25 d(+)‐glucose, 3 Na‐pyruvate, and 5 Na‐ascorbate (osmolality 305 mOsm; carboxygenated with 95%O_2_/5%O_2_).

### 2.4 Astrocyte Volume and Chloride Imaging

Slices were transferred to an imaging bath kept at 34°C with a continuous flow of standard aCSF, containing (in mM): 125 NaCl, 3 KCl, 1.2 NaH_2_PO_4_, 26 NaHCO_3_, 1 MgSO_4_, 2 CaCl_2_, 10 d(+)‐glucose (osmolality 305 mOsm; carboxygenated with 95%O_2_/5%O_2_). A custom‐built galvanometer‐based two‐photon laser scanning system was used to acquire z‐stack time series images of fluorescently labeled astrocytes (40× objective; 512 × 512 pixels/image; excitation wavelength 840 nm; acquisition rate at 1.79 Hz; emission split with 509 nm dichroic beamsplitter, filtered with 483/32 nm and 530/55 nm emission filters). High‐resolution z‐stacks (66.06 μm image width; 50 × 1 μm steps) were captured before time series recordings for baseline soma area measurements. To image swelling‐induced responses, z‐stacks were acquired with a 2‐min interval (132.96 μm image width; 25 × 2 μm steps). After five acquisitions in standard aCSF, high K^+^ aCSF was perfused in the bath to induce astrocyte swelling. High K^+^ aCSF (6 mM [K^+^]_o_) was made by adding 3 mM KCl and leaving out an equimolar amount of NaCl.

### 2.5 Image Analysis

Motion correction of z‐stack time series was done using the ‘Correct 3D Drift’ plugin in ImageJ. For astrocyte volume fraction (VF) measurements, fluorescence emission from the SC FRET donor Cerulean (F_Cerulean_) was used, since Cerulean fluorescence is unaffected by changes in intracellular ion concentration. VF was obtained as described (Henneberger et al. 2020), and normalized to averaged VF in standard aCSF. Recordings in which the basal VF was unstable (> 12.5% difference in VF during the second half of the baseline when compared with the averaged first half of the baseline), and astrocytes showing < 5% swelling upon high K^+^ application, were excluded from further analysis. RVD (%) is defined as (peak VF change − end VF change)/peak VF change × 100. To assess chloride dynamics, the fluorescence emission from the chloride‐sensitive SC FRET acceptor YFP (F_YFP_) was acquired. The SC FRET ratio is calculated using F_YFP_/F_Cerulean_. Values were normalized to the averaged SC FRET ratio in standard aCSF. Baseline soma area was obtained by median‐filtering z‐stacks with a 2‐pixel radius, followed by conversion to a binary mask using the Li thresholding method. A region of interest (ROI) was aligned around the soma and the mask area within the ROI was taken as astrocyte soma area.

### 2.6 Electrophysiology

Slices were prepared as described above for two‐photon imaging, but to visualize astrocytes, slices were incubated in Sulforhodamine 101 (SR‐101)‐containing (1.0–2.5 μM) holding solution at 35°C for 15 min and transferred to room temperature holding solution.

Slices were placed in a recording bath kept at 30°C (unless specified otherwise), mounted in an upright microscope with a constant flow of standard aCSF, and prepared as described above. Whole‐cell patch clamp recordings from SR‐101‐labeled astrocytes in Layer 1 of the primary visual cortex were made using a Multiclamp 700B amplifier and pCLAMP 10.7 software (Molecular Devices, USA). Recording borosilicate glass electrodes had a resistance of 3–5.5 MΩ. To record intrinsic electrophysiological properties (recordings at room temperature), pipettes were filled with an internal solution containing in mM: 140 KCl, 1.0 MgCl_2_, 0.5 CaCl_2_, 10 HEPES, 5 EGTA, 3 Mg‐ATP, and 0.3 Na_2_‐GTP (pH adjusted to 7.30 with KOH, osmolality 290 mOsm). The resting membrane potential (*V*_M_) was obtained in current clamp mode without holding current immediately after achieving whole‐cell configuration. Recordings in voltage‐clamp mode used a holding potential of −80 mV. Although precise measurement of membrane capacitance in astrocytes is challenging due to their low membrane resistance, apparent membrane capacitance was determined by manual whole‐cell capacitance compensation in pCLAMP 10.7 software. To measure membrane resistance (*R*_M_), voltage steps from −120 to +30 mV (*Δ*_step_ = 10 mV, 1 s step duration) were applied 3 min after whole‐cell configuration. *R*_M_ was calculated as the inverted slope of the steady‐state current–voltage (*I*–*V*) relationship. To correct for the contribution of the resistance contributed by the pipette tip, pipette resistance (determined before the experiment) was subtracted from *R*_M_.

To assess gap junction coupling through tracer diffusion, pipettes were filled with an internal solution containing in mM: 115 K‐gluconate, 10 Hepes, 4 KCl, 4 MgATP, 0.3 NaGTP, 10 K_2_‐phosphocreatine, 0.2 EGTA, and biocytin (5 mg mL^−1^) (pH adjusted to 7.30 with KOH, osmolality 290 mOsm). Astrocytes were kept in current clamp mode for exactly 30 min following the establishment of a whole cell recording to allow for biocytin diffusion, after which the patch‐clamp pipette was retracted, and the brain slice was rapidly fixated in 4% PFA. Only astrocytes that maintained a healthy hyperpolarized membrane potential for the entire recording duration were included for analysis. Processing of slices for histological assessment of gap junction coupling is described below.

To assess the functional coupling with neighboring astrocytes, recordings were done with a similar internal solution as used for assessment of intrinsic electrophysiological properties, but with the 140 mM KCl replaced with an equimolar amount of NaCl (Kiyoshi et al. 2018). Syncytial isopotentiality was assessed by recording *V*_M_ in current clamp mode for 10 min, starting immediately after achieving whole‐cell configuration. In experiments in which gap junctions were pharmacologically blocked, slices were kept in extracellular solution containing meclofenamic acid (MFA; 100 μM) for at least 30 min before the start of the recording.

For the recording of synaptically activated glutamate transporter currents (STCs) and K^+^ currents, pipettes were filled with an internal solution containing in mM: 115 K‐gluconate, 6 KCl, 4 Mg‐ATP, 0.3 Na_2_‐GTP, 10 Na‐phosphocreatine, 10 HEPES, 5 glucose (pH adjusted to 7.25 with KOH, osmolality 295 mOsm). To prevent neuronal postsynaptic currents, AMPA receptors, NMDA receptors, and GABA_A_ receptors were blocked by the addition of CNQX or NBQX (10 μM), D(L)‐AP5 (50 μM), and gabazine (5 μM) to the standard aCSF. STCs were evoked with a glass electrode with a broken tip filled with standard aCSF, placed in Layer 1 at ~80 μm from the recorded astrocyte. The intensity of the stimulation pulse was adjusted to induce a reliable STC response. Single pulses or single pulses alternated with 9‐ and 10‐pulse trains (100 Hz) were applied every 20 s, each repeated five times and averaged. The 10‐pulse STC was isolated by subtracting the 9‐pulse trace from the 10‐pulse trace. Decay kinetics of STCs and slowly decaying K^+^ inward currents were analyzed by fitting a mono‐exponential function in Igor Pro 8 (WaveMetrics, USA). Recordings were excluded if access resistance increased to > 20 MΩ.

### 2.7 Immunofluorescence Analysis of Astrocyte Gap Junction Coupling

To assess gap junction coupling following biocytin tracer diffusion in patch clamp experiments, slices were placed in 4% PFA immediately following recording and fixed for 24 h at 4°C. Next, slices were washed three times in PBS for 1 h at room temperature. Slices were incubated with a primary antibody against Glutamine Synthetase (1:1000, MAB302, Sigma Aldrich, USA) overnight at 4°C. The following day, slices were washed three times for 1 h at room temperature, followed by fluorescent labeling of the primary antibody. Biocytin was visualized using a secondary antibody against Biotin, Alexa Fluor 488 streptavidin (1:500. S11223, Invitrogen, USA). After washing with PBS, nuclear staining was performed with DAPI (1:1000, D1306, Invitrogen, USA) for 30 min. Slices were transferred to a glass slide and mounted with ProLong Glass antifade mountant (P36980, Thermo Fisher, USA). To accommodate the thickness of the slice, two 0.16‐mm‐thick coverslips were fixed to each side of the glass slide using superglue, serving as spacers.

Confocal imaging was performed on a Leica Stellaris 8 confocal microscope using a 20× objective. Z‐stacks were obtained spanning > 60 μm from the surface of the brain slice downward, with a step size of 1.25 μm. Biocytin‐positive astrocytes were manually counted using ImageJ software.

### 2.8 Statistics

For each experiment, the number of animals used and the total number of measurements (cells, slices) on which analysis is based are indicated by *N* and *n*, respectively. Statistical analysis was performed in GraphPad Prism 9 (GraphPad, USA). Statistical tests are stated in figure legends.

## 3 Results

### 3.1 *Glialcam*‐null Astrocytes In Situ Have a Defect in Volume Regulation

**FIGURE 1:**
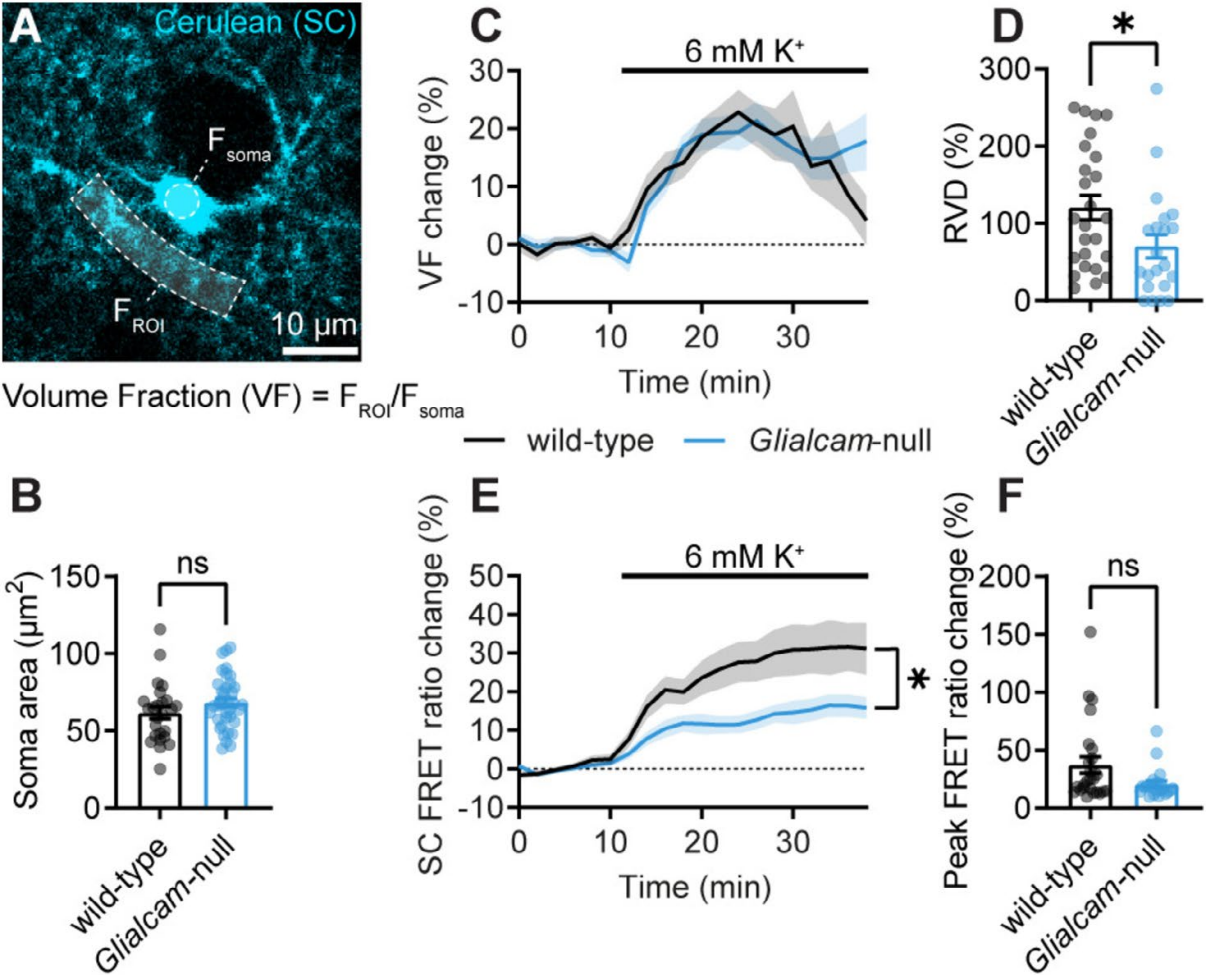
Astrocyte volume regulation upon increased bath [K^+^] is disrupted in *Glialcam*‐null mice. (A) The Cerulean fluorescence emission of SC in a wild‐type cortical astrocyte. (B) Astrocyte soma area is not significantly different in *Glialcam*‐null mice under standard conditions, indicating no chronic swelling of astrocyte somas (wild‐type: 61.63 ± 3.91 μm^2^, *N* = 5/*n* = 25 cells, *Glialcam*‐null: 68.27 ± 2.79 μm^2^, *N* = 4/*n* = 37, *p* = 0.160; unpaired *t*‐test). (C) Change in VF over time upon wash‐in of 6 mM [K^+^]_o_ reveals reduced RVD after swelling in *Glialcam*‐null astrocytes. (D) Quantification of RVD shows a significant decrease in mutants (wild‐type: 120.6% ± 15.85%, *N* = 4/*n* = 25, *Glialcam*‐null: 70.42% ± 15.15%, *N* = 4/*n* = 21, *p* = 0.029; unpaired *t*‐test). (E) The 6 mM [K^+^]_o_‐induced swelling is accompanied by an increase in the SC FRET ratio in the soma, indicating a decrease in intracellular [Cl^−^]. The traces are significantly different between genotypes, but post hoc analysis reveals no significant discovery at any time point (*p* = 0.030; two‐way ANOVA followed by Šídák's multiple comparisons test). (F) Quantification of the peak FRET ratio change does not show a significant change in [Cl^−^]_*i*_ dynamics in *Glialcam*‐null mice (wild‐type: 31.18% ± 6.76%, *N* = 4/*n* = 25, *Glialcam*‐null: 15.79% ± 2.78%, *N* = 4/*n* = 21, *p* = 0.090; Mann–Whitney *U*‐test). Data are plotted as mean ± SEM, together with dots for individual astrocytes in (B, D, and F).

Whether MLC astrocytes in situ have a defect in volume regulation, and what the accompanying intracellular chloride dynamics during swelling and volume regulation look like, is unknown. To investigate this, we simultaneously measured astrocyte volume changes and intracellular chloride ([Cl^−^]_i_) dynamics in acute brain slices. We used two‐photon imaging of astrocytes in the primary visual cortex that was virally transduced with the genetically encoded FRET‐based chloride sensor SC (Figure 1A) (Grimley et al. 2013). *Glialcam*‐null mice showed no difference in the baseline area of astrocyte somas, indicating that astrocyte somas are not chronically swollen (Figure 1B).

Previous studies that investigated astrocyte swelling during hypotonic or high K^+^ challenges have used diverse metrics to analyze volume dynamics. Studies monitoring the area of the soma and main processes of astrocytes did not reveal RVD upon cell swelling in intact tissue, while morphometric analysis of astrocyte fine processes is not possible because of imaging resolution. We therefore studied astrocyte volume dynamics by determining the astrocyte VF, a sensitive measure for the amount of neuropil occupied by astrocyte processes. We measured relative astrocyte volume changes in an ROI covering the fine astrocyte processes. The VF of these processes is defined as the ratio of fluorescence intensity from the chloride‐insensitive Cerulean donor fluorophore between an ROI covering the processes (F_ROI_) and an ROI covering the astrocyte soma (F_soma_), with the latter representing a VF of 100% (Figure 1A). Previously, it was shown that osmotic swelling increases the VF of astrocyte processes (Henneberger et al. 2020). We induced astrocyte swelling by increasing [K^+^]_o_ from 3 to 6 mM. In ~84% of astrocytes, this led to a robust increase in VF, indicating swelling of their processes, with an average peak VF increase of ~34.0% and ~33.6% in wild‐type and *Glialcam*‐null astrocytes, respectively (Figure 1C). Similar to the RVD process described in isolated astrocytes, the initial increase in VF was followed by a recovery of VF over time. Quantification of the amount of RVD (see Section 2) revealed that RVD was significantly reduced in *Glialcam*‐null mice (Figure 1D).

Chloride is a crucial ion in the RVD process (Jentsch 2016). The astrocyte swelling in response to increased [K^+^]_o_ and the following RVD process are therefore likely accompanied by changes in intracellular chloride concentration. To examine whether *Glialcam*‐null astrocytes have altered intracellular chloride dynamics during cell swelling and volume regulation, we measured the SC FRET ratio change in the soma. Upon astrocyte swelling, all astrocytes showed an increase in SC FRET ratio (Figure 1E), indicating a decrease in [Cl^−^]_i_. This decrease in [Cl^−^]_i_ persisted during the volume recovery phase in both wild‐type and *Glialcam*‐null astrocytes. The traces of SC FRET ratio change were significantly different between genotypes (Figure 1E), but post hoc analysis did not reveal significant differences at specific time points. On average, wild‐type astrocytes reached a higher SC FRET ratio change than *Glialcam*‐null astrocytes upon 6 mM [K^+^]_o_, but this difference was not statistically significant (Figure 1F). These results suggest that in the first ~10 min after high K^+^ application, [Cl^−^]_i_ is diluted by astrocyte swelling. Subsequent volume recovery in wild‐type astrocytes was not accompanied by a return to basal [Cl^−^]_i_. Since volume recovery reduces intracellular water content, it should lead to an increase in intracellular ion concentrations. The sustained decrease in [Cl^−^]_i_ therefore indicates a net Cl^−^ efflux from the cell during RVD. In contrast, *Glialcam*‐null astrocytes fail to show clear RVD. Therefore, the decrease in [Cl^−^]_i_ in *Glialcam*‐null astrocytes indicates sustained dilution of [Cl^−^]_i_. Taken together, *Glialcam*‐null astrocytes in intact brain tissue show a volume regulation defect, similar to the RVD defect previously observed in isolated *Mlc1*‐null primary astrocytes.

### 3.2 Intrinsic Electrophysiological Properties of *Glialcam*‐null Astrocytes

**FIGURE 2:**
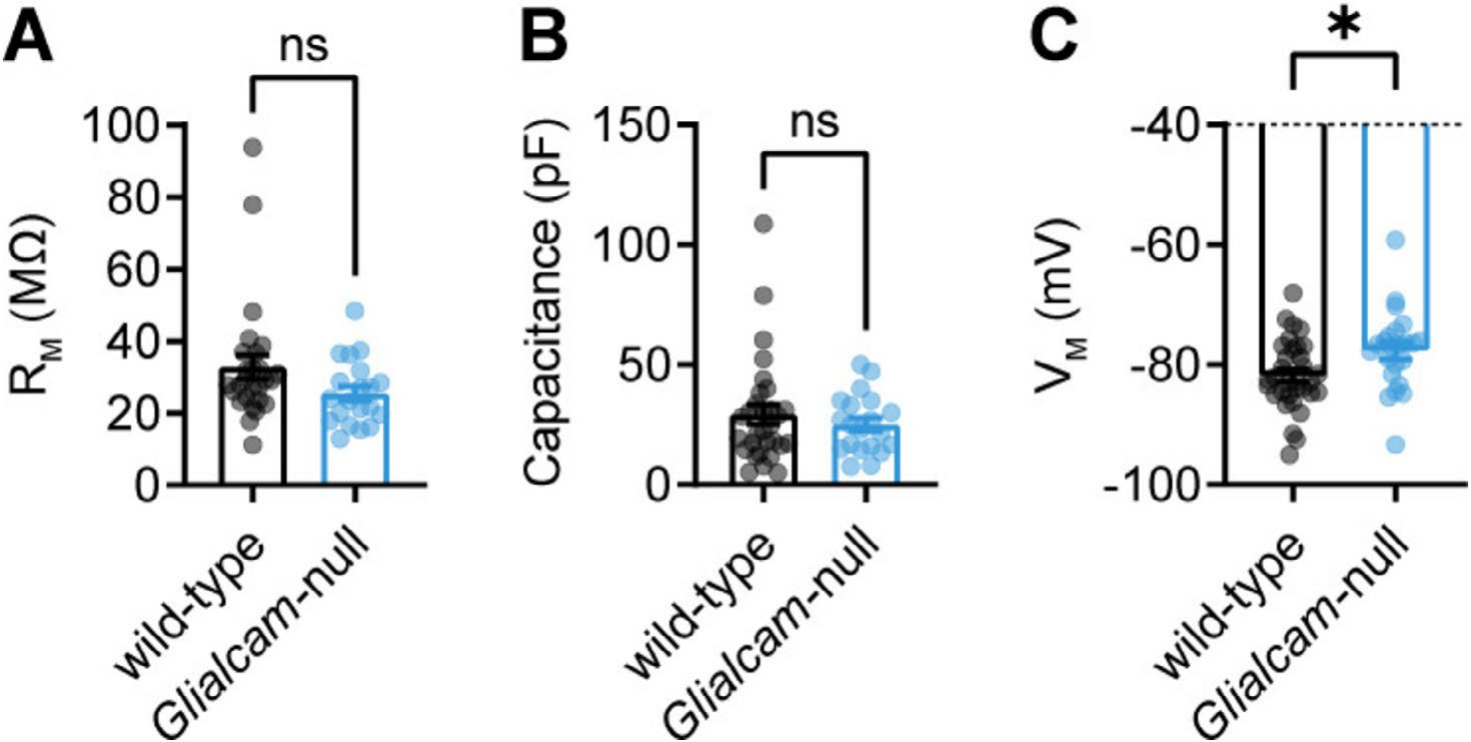
Intrinsic electrophysiological profile of cortical astrocytes is altered in *Glialcam*‐null mice. (A) Membrane resistance is normal in *Glialcam*‐null astrocytes (wild‐type: 32.91 ± 3.30 MΩ, *N* = 11/*n* = 27, *Glialcam*‐null: 25.50 ± 2.03 MΩ, *N* = 9/*n* = 20, *p* = 0.056; Mann–Whitney *U*‐test), suggesting overall membrane conductivity is not changed. (B) Membrane capacitance, reflecting membrane area, was not significantly different in Glialcam‐null astrocytes (wild‐type: 29.35 ± 4.07 pF, *N* = 12/*n* = 30, *Glialcam*‐null: 25.10 ± 2.72 pF, *N* = 8/*n* = 20, *p* = 0.818; Mann–Whitney *U*‐test). (C) The resting membrane potential of *Glialcam*‐null astrocytes was significantly depolarized compared to wild‐type astrocytes (wild‐type: −81.83 ± 1.015 mV, *N* = 13/*n* = 33, *Glialcam*‐null: −77.65 ± 1.385 mV, *N* = 10/*n* = 23, *p* = 0.016; unpaired *t*‐test). Data are plotted as mean ± SEM.

The homeostatic support by astrocytes depends on their unique electrophysiological properties (Zhou et al. 2021). To investigate whether *Glialcam*‐null astrocytes have altered intrinsic electrophysiological properties, we performed whole‐cell patch clamp recordings from astrocytes in acute brain slices. Wild‐type and *Glialcam*‐null astrocytes had a comparably low membrane resistance (Figure 2A), a characteristic feature of astrocytes that is due to their high K^+^ conductance. The capacitance of cells is proportional to the membrane surface. Although precise measurement in astrocytes is challenging due to their low membrane resistance, our recordings showed no significant differences in apparent capacitance between genotypes (Figure 2B). Several homeostatic functions of astrocytes depend on their highly hyperpolarized resting membrane potential (*V*_M_). The *V*_M_ of *Glialcam*‐null astrocytes was significantly depolarized compared to the wild‐type (Figure 2C).

### 3.3 Reduced Gap Junction Coupling, but Intact Syncytial Isopotentiality, in the Cortex of *Glialcam*‐null Mice

**FIGURE 3:**
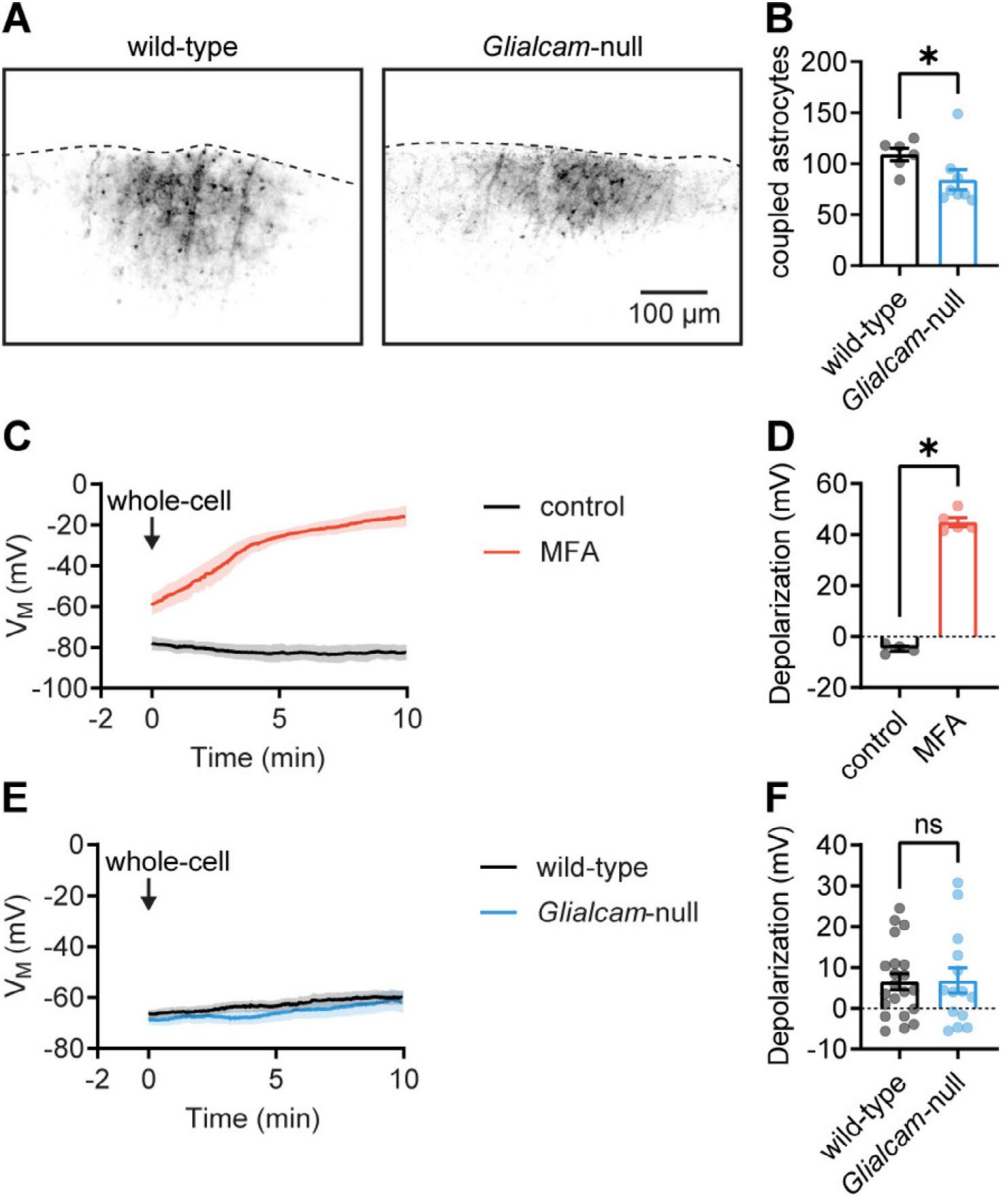
Astrocyte gap junction coupling is reduced, but syncytial isopotentiality is intact in *Glialcam*‐null mice. (A) Example images of wild‐type and *Glialcam*‐null cortical slices (V1) stained with Streptavidin 488 to detect biocytin after loading astrocytes for 30 min. (B) The number of coupled astrocytes was slightly, but significantly reduced in *Glialcam*‐null mice (wild‐type: 109.0 ± 6.028 neighbors, *N* = 2/*n* = 6, Glialcam‐null: 84.38 ± 9.937 neighbors, *N* = 2/*n* = 8, *p* = 0.040; Mann–Whitney *U*‐test). (C) Recording with a pipette solution in which K^+^ is replaced with Na^+^ pulls the membrane potential toward 0 mV. Because of electrical coupling to neighboring astrocytes in a syncytium, depolarization remains limited during the recording (black trace). In the presence of the gap junction blocker meclofenamic acid (MFA; 100 μM), the membrane potential is rapidly depolarized toward 0 mV (red trace). (D) Gap junction inhibition by 100 μM MFA significantly depolarized the membrane potential (control: −4.7373 ± 0.918 mV, *N* = 1/*n* = 4, MFA: 44.93 ± 1.763 mV, *N* = 2/*n* = 5, *p* = 0.016; Mann–Whitney *U*‐test). (E) Membrane potential recordings with Na^+^‐based pipette solution in wild‐type and *Glialcam*‐null astrocytes (F) 10 min after the start of recording, depolarization is similar between wild‐type and *Glialcam*‐null mice (wild‐type: 6.476 ± 1.947 mV, *N* = 6/*n* = 21, *Glialcam*‐null: 6.792 ± 3.105 mV, *N* = 4/*n* = 14, *p* = 0.928; unpaired *t*‐test), showing unchanged coupling strength. Data are plotted as mean ± SEM.

Astrocytes maintain homeostasis through extensive gap junction‐mediated coupling, forming an isopotential syncytium (Stephan et al. 2021). A recent study showed that conditional deletion of *Glialcam* from developing astrocytes impaired passive diffusion of a tracer molecule between adjacent astrocytes, suggesting a reduction in gap junction coupling (Baldwin et al. 2021). We performed a similar experiment in acute brain slices from *Glialcam*‐null mice. Whole‐cell patch‐clamp recordings from individual astrocytes were made with intracellular solution containing the tracer biocytin. After a loading period of 30 min, during which biocytin could diffuse through the astrocyte syncytium, slices were fixed, followed by immunofluorescence analysis of the number of biocytin‐positive astrocytes. This revealed a modest but significant reduction in gap junction coupling in *Glialcam*‐null mice (Figure 3A,B).

While tracer coupling is commonly used to assess gap junction coupling, it does not reflect the functional electrical coupling of astrocytes into an isopotential syncytium. This property can be assessed by measuring *V*_M_ in response to dialysis of high Na^+^ intracellular solution into the recorded astrocyte (Kiyoshi et al. 2018). In this paradigm, uncoupled cells rapidly depolarize toward 0 mV upon intracellular Na^+^ dialysis, whereas in astrocytes with strong electrical coupling, *V*_M_ remains hyperpolarized. In wild‐type slices, astrocytes maintained a hyperpolarized *V*_M_ during Na^+^ dialysis. In contrast, in the presence of the gap junction blocker MFA (100 μM), which disrupts the isopotential syncytium, astrocytes rapidly depolarized during Na^+^ dialysis (Figure 3C,D). Next, we used this method to assess functional electrical coupling in adult *Glialcam*‐null mouse brain slices. Both wild‐type and *Glialcam*‐null astrocytes showed limited depolarization upon Na^+^ dialysis and retained a quasi‐physiological *V*_M_ in these experiments (Figure 3E), indicating intact functional gap junction coupling. The amplitude of depolarization was not different between genotypes (Figure 3F), suggesting that functional astrocyte coupling strength is not altered. Taken together, these results indicate a slight reduction in gap junctional coupling in *Glialcam*‐null mice, which is not sufficient to impair the isopotentiality of the astrocyte syncytium.

### 3.4 *Glialcam*‐null Astrocytes Have Slower Glutamate Transporter Currents

**FIGURE 4:**
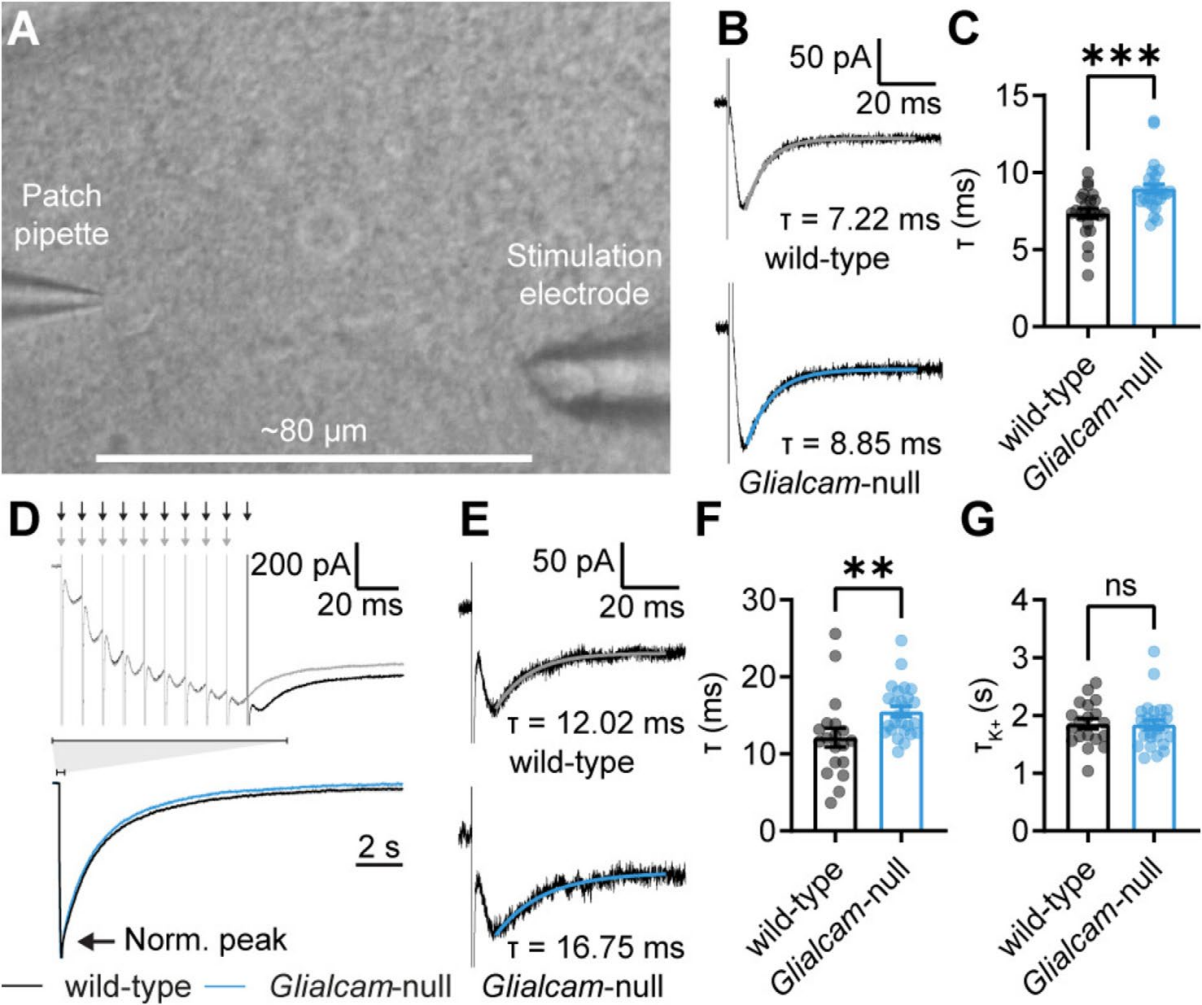
Glutamate uptake by cortical astrocytes is slower in *Glialcam*‐null mice. (A) Example patch clamp recorded astrocyte in V1 with extracellular electrode placed nearby to stimulate afferent fibers. (B) Representative current traces with fitted curve of the STC induced by single‐pulse stimulation. (C) The decay time of single pulse‐evoked STCs is significantly increased in *Glialcam*‐null astrocytes (wild‐type: 7.353 ± 0.321 ms, *N* = 10/*n* = 23, *Glialcam*‐null: 8.951 ± 0.289 ms, *N* = 15/*n* = 28, *p* = 0.0004; Mann–Whitney *U*‐test). (D) Top: Superimposed representative current traces upon 9‐ and 10‐pulse trains at 100 Hz in a wild‐type astrocyte. Bottom: Normalized and averaged slowly‐decaying inward K^+^ current evoked in wild‐type and *Glialcam*‐null astrocytes by 10‐pulse extracellular stimulation at 100 Hz, reflecting K^+^ uptake by astrocytes. (E) Representative traces with fitted curve of the 10th pulse STC, isolated by subtracting 9‐pulse traces from 10‐pulse traces. (F) The decay of 10th pulse‐evoked STCs is significantly slower in *Glialcam*‐null astrocytes (wild‐type: 12.12 ± 1.224 ms, *N* = 8/*n* = 19, *Glialcam*‐null: 15.53 ± 0.651 ms, *N* = 12/*n* = 26, *p* = 0.002; Mann–Whitney *U*‐test). (G) The decay time constant of the K^+^ current is not significantly different between wild‐type and *Glialcam*‐null astrocytes (wild‐type: 1.856 ± 0.087 s, *N* = 8/*n* = 19, *Glialcam*‐null: 1.844 ± 0.080 s, *N* = 12/*n* = 26, *p* = 0.920; unpaired *t*‐test). Data are plotted as mean ± SEM.

An important function of astrocytes is the uptake of extracellular glutamate and potassium, which helps control neuronal excitability. Glutamate transporters, such as the ubiquitous astrocyte transporter GLT‐1, are electrogenic. Therefore, the dynamics of astrocyte glutamate uptake can be assessed by measuring transporter currents using whole‐cell patch clamp (Capuani et al. 2016; Djukic et al. 2007; Romanos et al. 2020). To investigate potential alterations in glutamate uptake in *Glialcam*‐null mouse astrocytes, we recorded synaptically activated transporter currents (STCs). STCs were induced by single‐pulse electrical stimulation of synaptic transmission with an extracellular electrode in Layer 1 of V1, in the presence of synaptic receptor blockers (Figure 4A–C). The fast‐decaying component of the elicited current is the STC (Capuani et al. 2016; Romanos et al. 2020). The decay time constant of the STC was significantly increased in *Glialcam*‐null astrocytes (Figure 4B,C), indicating slower glutamate uptake. STC decay kinetics upon high‐frequency 10‐pulse stimulation at 100 Hz was also slower in *Glialcam*‐null astrocytes compared with wild‐type astrocytes (Figure 4D–F). During such prolonged stimulation, STC dynamics gradually become slower, indicating saturation of the glutamate uptake mechanism. This slowing down of the glutamate uptake was similar between genotypes, as no significant difference was found in the ratio between the decay time constants of high‐frequency and single‐pulse stimulation (data not shown). Therefore, the difference in glutamate uptake kinetics is not caused by faster saturation of glutamate uptake in *Glialcam*‐null mice. To assess whether the speed of K^+^ uptake is affected in astrocytes of *Glialcam*‐null mice, we quantified the time constant of the slowly decaying current after high‐frequency stimulation (Figure 4D). As astrocytes act as K^+^ bioelectrodes, this current reflects extracellular [K^+^] (Meeks and Mennerick 2007). The decay of the K^+^ current was not altered in *Glialcam*‐null astrocytes (Figure 4G), suggesting that K^+^ uptake is not slowed down. Taken together, glutamate uptake by astrocytes is slower in *Glialcam*‐null mice.

## 4 Discussion

Using acute brain slices from an established MLC mouse model, we studied astrocyte volume regulation and electrophysiology in the intact MLC brain. We show that volume regulation upon physiologically relevant cell swelling is disturbed in cortical *Glialcam*‐null astrocytes. This consolidates studies in isolated cells showing a similar RVD phenotype. By measuring astrocyte electrophysiological properties with whole‐cell patch clamp recordings, we show that *Glialcam*‐null astrocytes have a depolarized resting membrane potential, which is expected to impact the homeostatic regulation of extracellular ions and neurotransmitters. Tracer coupling experiments reveal a decrease in the amount of gap junction coupling between astrocytes, but this decrease is not sufficient to disrupt the electrical isopotentiality of the astrocyte syncytium. Finally, we show that astrocyte glutamate uptake following evoked synaptic activity is slowed in *Glialcam*‐null mice. Thus, astrocytes in MLC have multiple electrophysiological impairments.

### 4.1 Disrupted Astrocyte Volume Regulation in the MLC Brain

Disrupted astrocyte volume regulation is a hallmark of MLC. It was previously observed in patient‐derived lymphoblasts and cultured astrocytes as impaired RVD and linked to a defect in the opening of VRACs (Dubey et al. 2015; Ridder et al. 2011). Our experiments in acute brain slices confirm that intact MLC astrocytes show a similar RVD defect following physiologically relevant cell swelling.

Previous imaging studies on the dynamics of astrocyte volume in acute brain slices have used astrocyte soma size as a proxy for astrocyte volume, but RVD of soma size has not been observed (Florence et al. 2012; Walch et al. 2022, 2020). Since most astrocyte swelling in situ occurs in the fine processes of astrocytes, 3D confocal morphometry is a more sensitive approach to measuring astrocyte volume dynamics (Chvatal et al. 2007). This approach has allowed observation of RVD in a subset of astrocyte processes, while in other morphometric studies, no RVD was described (Kolenicova et al. 2020). Since a large fraction of fine astrocyte processes cannot be directly visualized using standard light microscopy, we used an alternative approach. We focused on volume changes in fine astrocyte processes by monitoring the astrocyte VF upon a modest increase in [K^+^]_o_ (from 3 to 6 mM). The VF parameter provides an easy way to resolve the amount of volume occupied by fine astrocyte processes and is sensitive to swelling‐inducing stimuli (Henneberger et al. 2020). Our results show that the VF can be used to measure astrocyte RVD in acute brain slices. Therefore, in line with earlier studies, we confirm that astrocyte volume regulation mainly takes place in fine processes rather than in the astrocyte soma (Chvatal et al. 2007).

We combined VF measurements with intracellular chloride imaging using SC. Both in wild‐type and *Glialcam*‐null astrocytes, initial astrocyte swelling is associated with an increase in the SC FRET ratio. This likely indicates a reduction in intracellular chloride due to chloride dilution. It should be noted that SC also shows pH sensitivity (Grimley et al. 2013). Intracellular alkalization leads to increased fluorescence of the YFP acceptor, which increases the FRET ratio. The change in extracellular potassium (3–6 mM) that we use to induce astrocyte swelling can lead to intracellular alkalization due to reverse activation of the Na^+^/HCO_3_^−^ transporter NBCe1. The expected magnitude of this alkalization is ~0.1 (Brookes and Turner 1994). The predicted effect of this alkalization on the SC FRET ratio (Grimley et al. 2013) is well below what we observe in our experiments. Therefore, the change in the SC FRET ratio likely mainly indicates a reduction in intracellular chloride. When the VF subsequently recovers in wild‐type astrocytes, intracellular chloride stays low. This suggests net chloride extrusion during VF recovery. Although a trend toward more chloride retention was observed in *Glialcam*‐null astrocytes during swelling, this did not reach significance. However, since *Glialcam*‐null astrocytes show no VF recovery, the fact that their chloride levels are similar to or higher than those in wild‐type astrocytes suggests reduced swelling‐induced chloride extrusion in these cells. This is in line with the observation of reduced swelling‐induced chloride currents due to disrupted activation of volume‐regulated anion channels in cultured cells with hampered *Glialcam* or *Mlc1* expression.

### 4.2 Altered Electrophysiology of MLC Astrocytes In Situ

We observe that cortical *Glialcam*‐null astrocytes are slightly depolarized, without a significant change in membrane resistance. The latter suggests that there are no major alterations in ion channel density. This is different from what is, for example, observed in conditional *K*_*ir*_ *4.1* knockout (cKO) mice, where strong depolarization of astrocytes is combined with a large increase in membrane resistance (Djukic et al. 2007). A potential explanation for the depolarized V_M_ in the absence of an altered membrane resistance is a disturbance in the ionic composition of the astrocyte cytosol in *Glialcam*‐null mice. Such a disturbance is hard to experimentally establish, but future experiments aimed at determining the ion composition of the astrocyte cytosol, using K^+^ or Na^+^ indicators, might answer this question.

Measurement of glutamate transporter currents reveals that glutamate uptake is slowed in *Glialcam*‐null astrocytes. This could be linked to the observed depolarization of *Glialcam*‐null astrocytes: depolarization can affect the functionality of glutamate transporters. The driving force for these transporters is the inward Na^+^ gradient, and their efficacy is higher when the membrane potential of astrocytes is more hyperpolarized (Zhou et al. 2021). Astrocyte depolarization inhibits the clearance of glutamate released upon neuronal activity and upon glutamate spot‐uncaging (Armbruster et al. 2022; Tyurikova et al. 2022). *K*_*ir*_ *4.1* cKO astrocytes also show reduced glutamate uptake (Djukic et al. 2007). Alternatively, potential changes at the tripartite synapse might underlie the glutamate uptake phenotype. GlialCAM shows a tight molecular interaction with MLC1, and loss of GlialCAM hampers MLC1 localization in astrocyte endfeet (Bugiani et al. 2017; Capdevila‐Nortes et al. 2013). We recently showed that MLC1 is also present in perisynaptic astrocyte processes (PAPs) (Kater et al. 2023). In *Mlc1*‐null mice, we found that PAP tips are retracted from excitatory synapses, leading to slower glutamate reuptake. The expression of GlialCAM in PAPs in CA1 has not been studied, but in the visual cortex, ~20% of intracortical excitatory synapses show colocalization with GlialCAM (Baldwin et al. 2021). It is possible that PAPs are also retracted from synapses in *Glialcam*‐null mice, resulting in slower glutamate uptake by astrocytes.

Our finding that astrocytic K^+^ uptake speed is intact in *Glialcam*‐null mouse brain slices is in line with experiments in which the dynamics of [K^+^]_o_ was measured in hippocampal brain slices using K^+^ electrodes. With this method, it was found that synaptic stimulation caused larger rises in [K^+^]_o_ in MLC mice, but that the decay time of [K^+^]_o_ recovery was unaffected (Dubey et al. 2018).

Studies on the interaction of GlialCAM with gap‐junction proteins prompted us to investigate tracer diffusion and electrical coupling of astrocytes in *Glialcam*‐null mice (Baldwin et al. 2021; Wu et al. 2016). Tracer diffusion experiments revealed a modest reduction in astrocyte gap junction coupling. However, loading astrocytes with [Na^+^] through the whole‐cell patch‐clamp pipette revealed no difference in the functional electrical coupling of *Glialcam*‐null astrocytes when compared to the wild‐type (Ma et al. 2016). Therefore, syncytial isopotentiality is intact upon loss of GlialCAM. This observation is in line with the suggestion that a minimum number of 7–9 gap‐junctional coupled astrocytes is required for achieving syncytial isopotentiality (Baldwin et al. 2021; Ma et al. 2016). The reported number of tracer‐coupled astrocytes greatly exceeds this minimum requirement. Thus, the reduction in spatial coupling following loss of GlialCAM likely has little effect on the electrical functioning of the astrocyte syncytium.

In conclusion, several properties of morphologically complex astrocytes are disturbed in the *Glialcam*‐null mouse model for MLC. This study helps to build a bridge between studies on relatively simple cell culture models and the complex dysfunction of astrocyte‐neuron networks in the intact brain.

## Author Contributions

S.K., R.M., M.S.v.d.K., and H.D.M. conceptualized the study and contributed to the interpretation of data. S.K., N.M., T.S.H., and R.M. contributed to the acquisition and analysis of data. R.M. acquired funding. S.K. and R.M. drafted the manuscript and prepared the figures. All authors provided critical feedback and approved the final version of the manuscript.

## Ethics Statement

All experiments were approved by the Animals Ethical Committee of the Vrije Universiteit Amsterdam or the Amsterdam University Medical Center in accordance with Dutch law.

## Conflicts of Interest

The authors declare no conflicts of interest.

## Data Availability

The data that support the findings of this study are available from the corresponding author upon reasonable request.
